# Plant-Based Functional Formulae Mitigate Western Diet-Induced MASLD by Modulating Hepatic Oxidative Stress, Inflammation as Well as Nrf-2/HO-1 and NF-κB Gene Expression

**DOI:** 10.3390/foods15193447

**Published:** 2026-09-27

**Authors:** Rasha S. Mohamed, Karem Fouda, Noha E. Ibrahim

**Affiliations:** 1 Department of Nutrition and Food Sciences, National Research Centre, Cairo 12622, Egypt; karemfouda@gmail.com; 2 Department of Microbial Biotechnology, National Research Centre, Cairo 12622, Egypt; nohaelsayed855@gmail.com

**Keywords:** MASLD, psyllium, dill, leek, Western diet

## Abstract

Psyllium has long been used to improve satiety and control weight. Leek and dill leaves are abundant in bioactive substances that have been confirmed to have anti-inflammatory and antioxidant properties. Our goal was to assess the hepatoprotective effects of two formulae (dill and psyllium or leek and psyllium) against oxidative stress and inflammatory markers in a metabolic dysfunction-associated steatotic liver disease (MASLD) rat model. Twenty four rats were used in this study. Rats were given a high-fat, high-sucrose diet (HF/HS), a Western diet, to create a MASLD model. The two formulae showed lower levels of serum alanine transaminase and aspartate transaminase, as well as a preventive effect against obesity, as shown by lower levels of leptin (with changes of 60.57 and 67.14%, respectively) and body weight gain (with changes of 25.57 and 29.04%, respectively). The two formulae resulted in a reduction in blood glucose, insulin, cholesterol, triglycerides, oxidative markers, and inflammatory markers upon feeding on the HF/HS diet. The histological evaluation in rats treated with the two formulae revealed reduced hepatic vacuolization and lobular inflammation. The two formulations reduced the mRNA expression of nuclear factor-kappa B (NF-κB) while increasing the mRNA expression of nuclear erythroid 2-related factor 2 (Nrf2) and heme oxygenase 1 (HO-1). The results indicated the potential efficacy of the two prepared formulations in alleviating and preventing MASLD in the rat model evaluated.

## 1. Introduction

The Western diet (WD) is low in fruits, vegetables, and whole grains and high in refined sugar and saturated fats. This food pattern is becoming more commonplace globally and has come to represent contemporary eating patterns in Western societies [1]. High-fat WD raises diacylglycerol and free fatty acids in skeletal muscle, hepatocytes, and fat cells [2]. More than 5% of hepatocytes have this buildup, which directly affects insulin signaling and contributes to fatty liver disease linked to MASLD. A significant clinical and financial burden, MASLD is one of the most prevalent chronic liver diseases worldwide, ranging from simple steatosis to steatohepatitis, fibrosis, cirrhosis, and hepatocellular cancer. MASLD impacts about 25% of adults worldwide [3]. The frequency of MASLD is rising along with the rates of obesity and type 2 diabetes worldwide. These non-communicable diseases cause 41 million deaths annually, or 74% of all deaths worldwide according to the World Health Organization [4]. Hepatic steatosis combined with one of the following metabolic abnormalities—obesity, type 2 diabetes mellitus, or at least two metabolic dysfunctions such as increased waist circumference, high blood pressure, hypertriglyceridemia, pre-diabetes, and insulin resistance—is diagnosed as MASLD [5]. Oxidative stress can be brought on by fat from a Western diet. Overindulgence in a high-fat WD causes the liver to undergo free fatty acid oxidation, which is linked to electron transfer in the electron transport system to produce ATP. When these products are combined with oxygen, reactive oxygen species (hydrogen peroxide and superoxide anion radicals) can build up and damage cells. Antioxidants work to counteract this process [6]. Furthermore, nuclear factor-kappa B (NF-κB) can be directly activated by fat from a Western diet, hence increasing the expression of pro-inflammatory cytokines. Since the anti-inflammatory and antioxidant defense system depends on nuclear erythroid 2-related factor 2 (Nrf-2), changes in Nrf-2 and NF-κB have a major impact on how MASLD progresses [7].

The amazing nutritional, functional, and health benefits of psyllium fiber, which is derived from the seeds of *Plantago ovata*, have attracted a lot of attention lately [8]. Psyllium is mostly soluble fiber that does not ferment or digest and, when wet, produces a thick gel. Psyllium gel slows the breakdown and absorption of nutrients in the small intestine by increasing the viscosity of chyme. Psyllium lowers blood cholesterol and significantly improves glycemic control [9].

Dill is a popular aromatic plant that grows extensively in Europe, Western Asia, and the Mediterranean region. Since ancient times, dill has been utilized for both culinary and medicinal purposes. Additionally, dill is a rich source of flavonoids and other phenolic compounds that have a number of therapeutic properties, such as anti-inflammatory, antidiabetic, antioxidant, anti-hypercholesterolemic and anticancer activities [10,11].

Leek, a popular food item, is also used in traditional Asian medicine to cure a range of ailments. Bioactive compounds found in leeks include sulfur compounds, fiber, steroid compounds, and flavonoids. Numerous studies have demonstrated the following effects of these active ingredients: blood circulation promotion, cholesterol reduction, fatigue relief, anti-inflammatory, cell metabolism regulation, anti-cancer, anti-oxidation, and blood sugar and fat reduction [12,13].

This study examined the efficacy of two plant formulations consisting of dill leaves and psyllium fiber or leek leaves and psyllium fiber in protecting against fat accumulation, oxidative stress, and the production of inflammatory cytokines that contribute to the development of MASLD caused by the Western diet. To the best of our knowledge, the present study is the first to investigate the effects of these plant-based formulations on the prevention of HF/HS-induced MASLD in a rat model.

## 2. Materials and Methods

### 2.1. Materials

Psyllium fiber, fresh dill leaves (*Anethum graveolens*, Apiaceae family) and fresh leek leaves (*Allium ampeloprasum*, Amaryllidaceae family) were purchased from the local market. Dill and leek were authenticated by Abdelmiged Ali Abedelmiged, professor of flora and plant taxonomy, Egypt Agriculture Research Center. DPPH (1,1-diphenyl-2-picrylhydrazyl) and 2,2-Azino-bis(3-ethylbenzothiazoline-6-sulfonic acid) diammonium salt (ABTS) were provided by Sigma (Sigma-Aldrich, St. Louis, MO, USA). Enzyme-linked immunosorbent assay (ELISA) kits were obtained from Sunlong Co., Ltd. (Shanghai, China).

### 2.2. Preparation of Functional Formulae

Fresh dill and leek leaves were thoroughly washed under running water, freeze-dried separately using a freeze dryer (ALPHA 1–4 LSC, Osterode, Germany) for 48 h at −52 °C, and then ground into a very fine powder. This fine powder was sieved and stored in bags at 4 °C until use. The first formula consisted of freeze-dried dill leaves and psyllium fiber in a 4:1 ratio (*w*/*w*), while the second formula consisted of freeze-dried leek leaves and psyllium fiber in a 4:1 ratio (*w*/*w*). This ratio was selected based on preliminary studies.

### 2.3. Scanning Electron Microscopy (SEM)

The morphological structure of the psyllium fibers was examined using high-resolution scanning electron microscopy (SEM) model (TESCAN VEGA 3 with a field emission gun, Brno-Kohoutovice, Czech Republic). A Quorum Q 150 ES (Quorum Technologies, Oxford, UK) was used to coat the samples with gold for sixty seconds. The SEM images are considered representative of the examined fiber samples.

### 2.4. Determination of the Swelling Ratio of Psyllium Fibers

According to Fouda and Mohamed [14], the swelling ratio was determined by measuring the initial volume of dry psyllium fibers following the addition of 0.1 g of fiber powder to a graduated cylinder with a 10 mL capacity. A thick gel developed after the required volume level was reached by adding deionized water. The swelling index was calculated using the following formula after the volume of the swollen fiber was measured after a full day.

Swelling ratio = V1/V0(1)

where V0 is the initial dry fiber volume, and V1 is the terminal volume after 24 h.

### 2.5. Phytochemical Profile of Freeze-Dried Dill and Leek

#### 2.5.1. Determination of Fatty Acids by GC-FID

Gas chromatography with flame ionization detection (GC-FID) was used to measure fatty acid contents using a slightly modified version of the method of Jarukas et al. [15]. A 5 g sample was homogenized in a hexane/isopropanol solvent combination (3:2 *v*/*v*) for 30 s in order to extract crude lipids. After centrifuging the homogenate for ten minutes at 10,000 rpm, the supernatant was moved to a reaction tube with a cover. An amount of 5 mL of 2% methanolic sulfuric acid was added to start the acid-catalyzed transesterification process. This was followed by vortex mixing and 12 h of incubation in a water bath at 50 °C. Five milliliters of 5% sodium chloride was used to quench the reaction once it had cooled to room temperature. Five milliliters of hexane was used to extract fatty acid methyl esters (FAMEs). To guarantee full phase separation, the organic phase was collected, neutralized with 5 mL of 2% potassium bicarbonate, and let to stand for 4 h. A Shimadzu GC-2010 Plus (Shimadzu Corporation, Kyoto, Japan) was used to quantify the separated FAMEs. The conditions used were as follows: 1 μL of injected volume; 1.26 mL/min of carrier gas helium; injector temperature of 230 °C; flame ionization detector temperature of 250 °C; split ratio of 1:20; and oven temperature ranging from 100 to 240 °C, with a stepwise temperature program over the 71.67 min run period. A 100 m Restek RT2560 column (Restek Corporation, Centre County, PA, USA) with a diameter of 0.25 mm and a thickness of 0.20 µm was used for the assay.

#### 2.5.2. Preparation of Dill and Leek Extracts for Phytochemical Profile

Two grams of each sample were placed in a 250 mL conical flask containing 10 mL of methanol in order to extract the freeze-dried dill and leek samples. The conical flask was then stirred with a magnetic stirrer for five minutes. The flask was sonicated for five minutes after being covered with tin foil. The solution was centrifuged (3000× *g*) for ten minutes. A 0.45 µm filter was used to filter the extracted materials [16].

#### 2.5.3. Determination of Vitamins by HPLC

According to Salah El-Din et al. [17], an Agilent 1260 series (Agilent Technologies, Santa Clara, CA, USA) was used for the HPLC analysis. an Agilent C18 (4.6 mm × 250 mm i.d., 5 μm) column was used for fat-soluble vitamins. The flow rate was 1.2 mL/min, and the mobile phase was methanol:acetonitrile 65:35. Each of the sample solutions had an injection volume of 20 μL. The DAD was set to 325 nm. The excitation/emission ratio of the fluorescence detector was set at 290/330 nm. The temperature of the column was kept at 40 °C.

ZORBAX SB-C8 (4.6 mm × 150 mm i.d., 5 μm) was used for the separation of water-soluble vitamins. The multi-wavelength detector was monitored at 280 nm. Water with 0.01% TFA (pH 2.9) (A) and methanol (B) at a flow rate of 1.5 mL/min made up the mobile phase. The injection volume was 5 µL, and the mobile phase was sequentially set in a linear gradient, as indicated in Table 1.

#### 2.5.4. Determination of Phenolic Compounds Profile by HPLC

Phenolic compounds, as mentioned previously [16], were analyzed using an Agilent 1260 series HPLC. The Zorbax Eclipse Plus C8 column (4.6 mm × 250 mm i.d., 5 µm) was used for the separation. Water (A) and 0.05% trifluoroacetic acid in acetonitrile (B) at a flow rate of 0.9 mL/min made up the mobile phase. The ordered linear gradient programming phases for the mobile phase were listed in Table 2.

The turning point for the multi-wavelength detector was 280 nm. Each sample solution had a 5 µL injection volume. The temperature of the column was kept at 40 °C. To determine each compound’s concentration, the peak area of the sample was compared to the corresponding standard (gallic acid, chlorogenic acid, catechin, methyl gallate, caffeic acid, syringic acid, pyrocatechol, rutin, ellagic acid, coumaric acid, vanillin, ferulic acid, naringenin, daidzein, quercetin, cinnamic acid, rosmarinic acid, kaempferol, and hesperetin).

#### 2.5.5. Determination of Total Phenolic Content

The Folin–Ciocalteu method was used to calculate the phenolic concentrations, which were then represented as gallic acid equivalents (GAEs) per gram of material based on Singleton et al. [18].

#### 2.5.6. Determination of Total Flavonoid Content

According to Campos et al. [19], the aluminum chloride colorimetric test was utilized to determine the total flavonoid concentration. The total flavonoid concentration was determined and expressed as milligrams of catechin equivalent (mg CE) per gram of sample using a calibration curve made with catechin.

#### 2.5.7. Determination of Antioxidant Activity

FRAP (ferric ion reducing antioxidant power), ABTS radical cation test, and DPPH radical scavenging assay were carried out.

The DPPH radical scavenging assay was carried out in accordance with Brand-Williams et al. [20]. The proportion of DPPH radical scavenging activity was calculated using the following formula:

DPPH radical scavenging activity (%) = (Ac − As/Ac) × 100(2)

where As is the absorbance of the sample, and Ac is the absorbance of the control without the sample.

The procedure outlined by Prior et al. [21] was followed while performing the ABTS radical cation assay. The proportion of ABTS scavenging activity was determined using the following formula:

ABTS scavenging (%) = (Ac − As/Ac) × 100(3)

where As is the absorbance of the sample, and Ac is the absorbance of the control without the sample.

The FRAP test was performed in compliance with Gomez et al. [22]. Plotting the absorbance at 593 nm could create a calibration curve with various FeSO_4_ concentrations.

### 2.6. Animal Study

#### 2.6.1. Experimental Animals

The Medical Research Ethics Committee (MREC) of the National Research Centre in Egypt approved this study’s methodology and ensured that each animal was treated humanely (Code 8497082023). Every technique is reported in compliance with ARRIVE guidelines. Twenty four healthy, six-week-old male Wistar albino rats (*Rattus norvegicus*) weighing 133.58 ± 4.21 g (mean ± SD) were purchased from the National Research Centre’s animal house in Egypt. Rats were housed in a 12 h light/dark cycle with controlled humidity (50 ± 10%) and temperature (22 ± 2 °C). All rats had unlimited access to food and water during the experiment.

#### 2.6.2. Diets

The animals were fed one of two diets (Table 3): a balanced diet made as described by Reeves et al. [23] or a high-fat, high-sucrose (HF/HS) diet, as a Western diet, made in accordance with Yang et al. [24].

#### 2.6.3. Groups and Treatments

Twenty-four rats were randomly assigned to four groups (*n* = 6) and given different treatments in compliance with the schedule, following a week of acclimatization to laboratory settings.

1. The normal control group (CN) received a balanced diet and five milliliters of distilled water each day for 8 weeks.

2. The Western diet group (WD) received 5 mL of distilled water orally daily and was fed HF/HS diet for 8 weeks [24].

3. The third group (D-P) was fed HF/HS diet and given a daily dose of the functional formula one (2 g of freeze-dried dill and 0.5 g of psyllium fiber) dissolved in 5 mL of distilled water by gavage for 8 weeks.

4. The fourth group (L-P) was fed HF/HS diet and given a daily dose of the functional formula two (2 g of freeze-dried leek and 0.5 g of psyllium fiber) dissolved in 5 mL of distilled water by gavage for 8 weeks.

The total amount of food consumed and the amount of weight gained were computed at the end of the trial. Blood was obtained by retro-orbital sinus hemorrhage from rats that had been adequately anesthetized with sodium pentobarbital (50 mg/kg, i.p.). Following profound anesthesia, the animals were killed by cervical dislocation, and the livers were dissected. The blood samples were centrifuged for 15 min at 3500 rpm to extract the serum, which was then kept at −80 °C until analysis. For the histological examination, a section of the liver was submerged in formalin-saline (10%). In a cold potassium phosphate buffer (100 mM, pH 7.4), a different piece of the liver was homogenized (10% *w*/*v*). After centrifuging the homogenized samples, the supernatants were gathered and utilized for biochemical examination.

#### 2.6.4. Biochemical Analysis

The fasting blood glucose (FBG) was determined in a drop of blood from the tail using Accu-Chek Active (Accu-Chek, Mannheim, Germany). Using the colorimetric techniques, the serum of each rat was assayed for total antioxidant capacity (TAC), total cholesterol (Ch), triglycerides (TG), LDL-cholesterol, HDL-cholesterol, and the activities of alkaline phosphatase (ALP), alanine transaminase (ALT), and aspartate transaminase (AST) (Koracevic et al. [25]; Watson [26]; Megraw et al. [27]; Schriewer et al. [28]; Burstein et al. [29]; Bessey et al., [30]; Reitman and Frankel [31]). Using the colorimetric techniques outlined by Nishikimi et al. [32], Sedlak and Lindsay [33], and Ohkawa et al. [34], the levels of liver superoxide dismutase (SOD) activity, reduced glutathione (GSH), and malondialdehyde (MDA) were measured.

#### 2.6.5. Determination of Hepatic Lipids

The Folch et al. [35] method was used to extract and weigh total hepatic lipids. To extract lipids, liver tissues (1 g) were homogenized in 5 mL of a 2:1 chloroform:methanol combination. The lipids were extracted by separating the top layer and evaporating the solvents after adding a standard saline solution (5 mL) to create two layers.

#### 2.6.6. Enzyme-Linked Immunosorbent Assays

Serum insulin (Cat No. SL0373Ra), serum leptin (Cat. No. EL0003Ra), liver tumor necrosis factor alpha (TNF-α, Cat No. SL0722Ra), liver interleukin-6 (IL-6, Cat No. SL0411Ra), liver interleukin 1-beta (IL-1β, Cat No. SL0402Ra), liver reactive oxygen species (ROS, Cat No. SL1189Ra), and liver toll-like receptor 4 (TLR4, Cat No. SL0699Ra) were all measured using Elisa kits (Sunlong Co., Ltd., Shanghai, China) in accordance with the manufacturer’s instructions.

#### 2.6.7. Quantitative Real Time-Polymerase Chain Reaction (qRT-PCR) for Investigation of Nrf-2, HO-1 and NF-κB mRNA Expression

Total RNA was isolated from 50 mg of rat liver using the RNeasy mini Kit (Qiagen; Germantown, MD, USA; Cat No. 74104) in accordance with the manufacturer’s instructions and kept at −20 °C. Absorbance at 260 nm (Thermo Scientific, Waltham, MA, USA) was measured using a Nanodrop Spectrophotometer to determine the RNA content and purity. Nrf-2, HO-1, and NF-κB gene expression was assessed in the presence of the housekeeping gene β-actin (forward and reverse primer sequences are detailed in Table 4). For one-step RT-PCR quantification, the QuantiTecht SYBR green Master Mix (Qiagen, MD, USA; Cat. No. 204243) was utilized. The reaction was carried out using the Stratagene Mx3000 P QPCR device (Agilent Technologies, Santa Clara, CA, USA). In short, 25 μL of 2 × SYBR green Master Mix, 0.5 μL of quantitect RT mix, 200 ng of each primer, and 4 μL of extracted RNA were added to a 50 μL reaction volume. The RT-PCR software’s thermal cycle was as follows: 50 °C for 30 min, 95 °C for 15 min, 94 °C for 15 s, annealing at 57 °C for 20 s, and lastly extension at 72 °C for 15 s for 40 cycles. The comparative thermal cycle (2^−ΔΔCT^) approach was utilized to determine the relative expression of the current amplified genes [36].

#### 2.6.8. Histopathological Examination

The initial two-step fixation involved immersing the liver tissue in 10% buffered formalin for two hours each, and then removing the fixative with distilled water for thirty minutes. The tissues were then subjected to a graded sequence of alcohol concentrations (70%, 90%, and 100%) in order to dehydrate them. The tissue was first subjected to 70% alcohol for 30 min, then 90% alcohol for an hour, and finally two one-hour cycles of pure alcohol. After dehydration, the samples were cleared using several xylene modifications. Tissue was submerged in a combination of 50% alcohol and 50% xylene for an hour, and then it was submerged in pure xylene for 1.5 h. After being soaked with hot paraffin wax, the samples were blocked out and embedded [37]. Hematoxylin and eosin, the standard staining method, were applied to paraffin sections (4–5 um).

### 2.7. Statistical Analysis

SPSS version 21 (SPSS Inc., Chicago, IL, USA) was used for the statistical analysis. After verifying the normal distribution of the data using the Shapiro and Levene tests, parametric tests were used for the statistical analysis. The results of the phytochemical profiles of leek and dill (*n* = 3), which were displayed as mean ± standard deviation (SD), were statistically evaluated using the independent-samples *t*-test. One-way analysis of variance (ANOVA) was used to statistically assess the in vivo assay data (*n* = 6), and the findings were presented as mean ± standard error of mean (SEM). The statistical differences between the groups were examined using the Duncan test. *p* < 0.05 was used to assess whether the differences were statistically significant.

## 3. Results and Discussion

### 3.1. The Scanning Electron Microscope and Swelling Ratio of Psyllium Fiber

The scanning electron microscope (SEM) is a highly useful tool for examining morphological structure; therefore, it was used in the present study to ensure the purity and characterize the surface structure of psyllium fibers, a feature that likely contributes to their physical properties. SEM of psyllium fibers (Figure 1) reveals the morphology of dense, longitudinally oriented fibers packed in a quasi-regular arrangement. The great capacity of psyllium fiber to absorb water is demonstrated by its swelling ratio of 5.3 ± 0.03 in neutral, deionized water. According to Strkalj et al. [38], when psyllium husks are combined with deionized water, their molecular chains entwine and form a three-dimensional lamellar structure with pores that allow water molecules to enter more easily. Water molecules also form hydrogen bonds with the hydroxyl groups of psyllium husks, demonstrating good hydrophilic ability. Stomach distension brought on by psyllium fiber swelling results in the production of satiating hormones and a sense of fullness [39]. Psyllium is utilized for weight loss and management because of its satiety properties [9].

### 3.2. Phytochemical Profile of Freeze-Dried Dill and Leek

Gas chromatography (GC-FID) analysis results (Table 5) revealed 17 distinct fatty acids identified in both dill and leek. Palmitic, stearic, linoleic, and linolenic fatty acids were the major fatty acids in both dill and leek. Together, they accounted for 85.73% and 90.22% of the total fatty acids in dill and leek, respectively. The proportion of oleic acid was relatively higher in dill (4.56%) compared to leek (2.47%). Conversely, leek exhibited a relatively higher proportion of linoleic and linolenic acids compared to dill. A trace proportion of nervonic acid was also detected in leek.

Dill and leek leaves are low in fat and provide few calories; furthermore, regarding the fat content, alpha-linolenic acid (omega-3) and linoleic acid (omega-6) constitute a large proportion of the fatty acids, according to the results (Table 5). Hegazy et al. [39] reported that linoleic and linolenic fatty acids are vital polyunsaturated fatty acids that have a significant impact on the onset and course of MASLD. While a higher dietary intake of omega-3 or a lower omega-6/omega-3 ratio helps prevent steatohepatitis and fibrosis, a raised ratio of omega-6 to omega-3 encourages inflammation and fat accumulation in the liver. Accordingly, the higher proportion of linolenic acid relative to linoleic acid in both plants makes them nutritionally desirable.

Table 6 presents the results regarding the vitamin content of both dill and leek. Regarding fat-soluble vitamins, vitamins A and E were quantified. The results showed that dill contained a higher concentration of vitamin A (25.9 µg/g) compared to leek (13.73 µg/g), whereas dill contained no vitamin E, unlike leek, which showed a concentration of 5.43 µg/g of plant material. As for the water-soluble vitamins (B-complex), the levels of thiamine, riboflavin, niacin, pyridoxine, and folate were determined; all were found to be present in significantly higher concentrations in dill compared to leek. Based on earlier studies that showed the beneficial function of the determined vitamins in preventing MASLD and related risks, these vitamins were assessed in dill and leek in the current study. Wu et al. [40] state that vitamins are generally necessary for controlling inflammation and hepatic metabolic processes, as well as for reducing liver fibrosis brought on by a high-fat diet. Additionally, vitamin E may have protective benefits against MASLD. According to Migni et al. [41], vitamin A is crucial for maintaining the integrity of the gut barrier, promoting epithelial immunity and reducing inflammation that could upset the gut–liver axis and raise the risk of MASLD. Vitamin A’s hepatoprotective effects include indirect antioxidant and anti-inflammatory effects across liver cell types, as well as nuclear receptor-mediated control of insulin function and lipid metabolism. As a result, clinical evidence consistently links vitamin A deficiency to the advancement of MASLD, associating lower levels of retinoic acids with more severe illness and mortality from fibrosis [42]. According to Li et al. [43], consuming more folate, choline, vitamin B1, and vitamin B2 may help prevent fatty liver degeneration. Numerous physiological activities in the body, such as nucleic acid and protein synthesis, amino acid balance, redox defense, methylation modification, and immunological response, depend on one-carbon (1C) metabolic reactions mediated by folate [44]. It has been shown that folate influences oxidative stress, chronic inflammation, and lipid metabolism in the liver, all of which contribute to fatty liver disease [45]. Furthermore, patients with MASLD who have low serum levels of the vitamin folate have a considerably higher chance of dying from all causes.

Of the 19 phenolic standards injected into the HPLC device, 18 phenolic compounds were detected in both dill and leeks. According to the results (Table 7), the most prevalent phenolic compounds in dill were chlorogenic acid (7370.94 µg/g), catechin (1528.47 µg/g) and gallic acid (878.39 µg/g) while the main phenolic components in leek leaves were found to be rutin (927.46 µg/g), rosmarinic acid (811.30 µg/g), and querectin (575.53 µg/g).

Wasli et al. [46] identified phenolic constituents in dill leaves, including chlorogenic acid, caffeic acid, ferulic acid, quercetin, and kaempferol. The study by Mahmoud et al. [47] found phenolic compounds in leek, including quercetin, apigenin 7-O-glucoside, quercetin-7-O-rutinoside, kaempferol 3-glucoside, gallic acid, and chlorogenic acid. The study linked these compounds to the leek’s neuroprotective effect against depression, which is caused by oxidative stress and elevated pro-inflammatory cytokines. Because of their strong antioxidant properties, most phenolic acids have exceptional bioactivity. Additionally, by controlling the composition of the intestinal flora, preventing inflammatory reactions, and controlling lipid metabolism, these substances have a significant impact on MASLD. Phenolic acids’ chemical structures are inextricably linked to their pharmacologic activity, especially their anti-inflammatory and antioxidative properties. Common phenolic acids have some chemical and biological characteristics due to their very similar structures [48]. According to Tauil et al. [49], numerous previous studies have revealed the beneficial effect of certain phenolic compounds (catechin, chlorogenic acid, kaempferol, and quercetin) found in dill and leeks in alleviating MASLD. By lowering hepatic fat accumulation, lipotoxicity, and oxidative damage, some phenolic substances, such as caffeic acid, can mitigate MASLD [50].

Table 8 demonstrates that dill had greater antioxidant activity than leeks, as evidenced by the significantly higher TPC, TFC, DPPH, ABTC, and FRAP values for dill. The antioxidant activity of leeks and dill can be primarily attributed to their content of bioactive compounds with antioxidant effects, such as flavonoids and phenolic compounds [11,51]. Biernacka et al. [51] identified strong connections between the phenolic content and ABTS and DPPH for leek and also noted that the method used to dry the green leaves affects antioxidant activity, explaining that freeze-drying, the method employed in our study, yields greater antioxidant activity than other drying techniques. According to Svobodová et al. [52], MASLD is closely associated with oxidative stress, which causes cellular lipotoxicity, mitochondrial malfunction, and endoplasmic reticulum stress. The harmful consequences of oxidative stress are often accompanied by changes in antioxidant defense mechanisms; thus, compounds with high antioxidant activity can help alleviate and prevent MASLD. Accordingly, the antioxidant activity of both plants may contribute positively to preventing and alleviating MASLD.

### 3.3. Findings of the Animal Study

#### 3.3.1. Effect of Functional Formulae on the Growth Performance

The data (Table 9) show that rats fed the WD diet had significantly (*p* < 0.05) higher total food intake, fat weight, and liver weight than normal rats. Rats in the WD group gained significantly (*p* < 0.05) more body weight than normal rats. In contrast, the rats on the WD diet that were given the dill-psyllium or leek-psyllium formula displayed a remarkable (*p* < 0.05) diminution in body weight gain, total food intake, fat weight, and liver weight when compared to the WD group.

The risk of MASLD is increased in people who have obesity, hyperlipidemia, hyperglycemia, insulin resistance, inflammation, and oxidative stress [53]. Due to their numerous targets and pathways, minimal side effects, and ability to reduce oxidative stress and inflammation, natural products, particularly plant-based natural products, have garnered significant interest in the treatment of MASLD [54]. Based on psyllium fiber’s ability to reduce fat absorption and promote weight loss, as well as the antioxidant and anti-inflammatory qualities of both dill and leeks, the current study assessed the potential efficacy of two formulae against MASLD: the first was made up of psyllium fiber and freeze-dried dill leaves, and the second was made up of psyllium fiber and freeze-dried leek leaves.

In the current study, rats fed a HF/HS diet gained weight in a similar manner to that reported by Zakaria et al. [55] who found that the high-fat diet led to weight gain in the rats, whereas phenolic compounds appeared to help mitigate this gain. Consequently, the phenolic content of dill and leeks likely contributed to preventing weight gain in the groups treated with both formulae, as evidenced by the study results (Table 9). Additionally, Lin et al. [56] reported that rats fed a HF/HS diet had much lower total food intake and greater body weight gain than normal rats. The HF/HS diet may exacerbate lipid metabolism-related conditions such as hyperlipidemia, atherosclerosis, insulin resistance, and elevated visceral fat. An imbalance between energy consumption and expenditure produces weight gain by causing fat to accumulate in white adipose tissue [57]. Psyllium fibers may play a positive role in reducing body weight, as well as liver weight and the amount of fat stored in the liver. Psyllium has long been used as a component or supplement to help regulate weight and increase satiety. Its main actions of water absorption and viscosification are significant because they raise the viscosity of chyme, a mixture of partially digested food and gastric juice, which in turn sets off several satiety-related processes. High chyme viscosity slows down enzyme-macronutrient interactions, which prevents the macronutrients from being broken down and allows them to pass through the small intestine more intact. Two physiological effects result from the prolonged presence of foods in the small intestine: the stimulation of the ileal brake mechanism and the release of hormones that control appetite. Hormones that control appetite act on the neurological system to increase feelings of fullness and decrease hunger. When more undigested nutrients enter the distal ileum, the ileal brake mechanism is activated. To maximize nutrient absorption and digestion, a distal-to-proximal feedback mechanism is activated, slowing the flow of a meal through the digestive system. Decreased jejunal motility, postponed gastric emptying, and decreased production of pancreatic enzymes, bile acids, and gastric acid are some of the cascade consequences that the ileal brake can produce, all of which contribute to the longer small intestine transit time. Increased satiety and decreased energy intake are the outcomes of all these systems, which eventually lead to weight control [9].

#### 3.3.2. Effect of Functional Formulae on Blood Glucose, Insulin, Leptin as Well as Liver Functions and Fat

The data (Table 10) show that rats fed the WD diet had significantly higher levels (*p* < 0.05) of FBG, insulin, leptin, AST, ALT, ALP, and liver fat than normal rats. Administration of the dill-psyllium or leek-psyllium formula resulted in significantly decreased (*p* < 0.05) levels of FBG, insulin, leptin, AST, ALT, ALP, and liver fat. The development of MASLD is more dependent on the quality of carbohydrates than on total intake. Hepatic de novo lipogenesis, increased visceral fat, worsened insulin sensitivity, and activation of inflammatory and fibrogenic pathways are all caused by excess refined carbohydrates and added sugars [58]. Our results are consistent with a study by Moreno-Fernández et al. [59], which discovered that rats fed a HF/HS diet had considerably higher levels of FBG, insulin, and leptin than normal rats. This study also found a strong correlation between oxidative stress and abdominal adiposity and impaired glucose metabolism, which in turn causes insulin resistance and the eventual progress of type 2 diabetes. In particular, the continuous liberation of free fatty acids (FFAs) stimulates the liver’s production of VLDL and cholesterol and stimulates gluconeogenesis. This leads to a disturbance in insulin signaling and glucose metabolism, ultimately resulting in insulin resistance [60].

Psyllium fibers, which also control digestion and intestinal absorption, may be involved in the blood sugar-lowering effects of the two formulae because they either improve insulin sensitivity or alter insulin secretion. Propionate’s inhibition of hepatic cholesterol production and indirect effects involving decreased glucose absorption are two further mechanisms that might be involved [61]. Significant hypoglycemic effects may also be attributed to dill leaves, which lower serum insulin levels, lower fasting blood glucose, and improve insulin resistance by reducing oxidative stress and advanced glycation end products [62]. According to Al-Khalaifah et al. [63], leek’s hypoglycemic effects could be explained by allyl-propyl-disulfide chemicals, which increase free insulin by competing with insulin for metabolism. Other theories include the production of antioxidant activity by phenolic acids or sulfur-containing substances, such as dialkyl disulfides, whose oxidized thiols can trap electrons from the body to function as antioxidants. Elevated serum liver enzymes are among the earliest signs of liver disease, and the ability of both formulations to suppress this elevation may indicate their efficacy in preventing liver disease.

In MASLD, fat accumulates in the liver, followed by oxidative stress and inflammatory cytokines. Abnormal lipid deposition in the hepatocytes is caused by an imbalance in hepatic lipid metabolism, which includes a disruption in fatty acid intake, synthesis, lipolysis, and fatty acid oxidation [64]. The current study showed increased levels of liver fat in the WD group. The effect of both formulae in reducing liver fat can be attributed to their bioactive compounds, particularly phenolic compounds, which prevent lipogenesis, or the synthesis of fat in the liver, and promote β-oxidation of fatty acids by influencing sterol regulatory element-binding protein-1c and acetyl-CoA carboxylase. Phenolic compounds also aid in preventing the oxidation of LDL-cholesterol [65].

#### 3.3.3. Effect of Functional Formulae on the Lipid Profile

The results (Table 11) showed that rats fed the WD diet had considerably (*p* < 0.05) higher levels of Ch, TG, LDL-Ch, non-HDL-Ch, VLDL, and the Ch/HDL ratio than the normal rats. The WD group’s HDL-Ch levels were significantly (*p* < 0.05) lower than those of normal rats. Rats fed the dill-psyllium or leek-psyllium formula while on the WD diet showed a remarkable (*p* < 0.05) elevation in HDL-Ch levels and a considerable (*p* < 0.05) diminution in LDL-Ch, non-HDL-Ch, VLDL, and Ch/HDL ratio levels.

According to a study by Moreno-Fernández et al. [59], rats fed a HF/HS diet had significantly higher levels of Ch, TG, and LDL-Ch than did normal rats. The study also found that surplus energy is stored in white adipose tissue during obesity in the form of fatty acids and TG, which causes adipocyte hypertrophy. These findings are consistent with the present results. Ectopic fat buildup results from the overproduction of adipocytes, which release a surplus of fatty acids back into the systemic circulation.

Psyllium fiber, dill, or leek is involved in the ability of the dill-psyllium or leek-psyllium formula to manage the lipids parameters. Viscous fiber’s capacity to bind bile acids and increase fecal excretion is assumed to be the cause of its fat-lowering benefits. Additionally, psyllium fiber encourages intestinal fermentation, which raises SCFA synthesis and alters cholesterol metabolism [66]. According to Sadeghi et al. [67], who investigated the beneficial effects of dill on lipid profiles, d-limonene and antioxidants, including polyphenols, vitamins, and carotenoids in dill, may be responsible for the anti-hyperlipidemic mechanism. Antioxidants aid in the defense against free radicals and oxidative damage. Endothelial dysfunction, which increases vulnerability to atherosclerosis, hypertension, and hypercholesterolemia, may be exacerbated by free radicals. Additionally, free radicals may speed up the atherosclerotic process and raise the oxidative load of LDL. Other possible explanations for dill’s hypolipidemic action include reduced cholesterol absorption through bile acid binding, suppression of fatty acid and cholesterol synthesis through suppression of acetyl-CoA carboxylase and HMG-COA reductase activity, and stimulation of cholesterol clearance through increased LDL receptors. High concentrations of polyphenols, antioxidants, and sulfur-containing substances like S-methylcysteine sulfoxide may be the cause of leek’s hypocholesterolemic and hypolipidemic effects [63].

#### 3.3.4. Effect of Functional Formulae on the Oxidative Stress

Rats fed the WD diet had significantly (*p* < 0.05) higher levels of liver MDA and ROS than normal rats, according to the results (Table 12). Serum TAC, liver SOD, and liver GSH values in the WD group were significantly (*p* < 0.05) lower than those in normal rats, most likely as a result of increased reactive oxygen species (ROS) production. Rats fed either the dill-psyllium or the leek-psyllium formula, on the other hand, exhibited significantly (*p* < 0.05) lower levels of MDA and ROS but higher values of TAC, SOD, and GSH.

These data are in line with a report by Hayakawa et al. [68] that identified inflammation and oxidative markers in rats fed high-fat, high-sucrose diets. Overindulgence in energy supplies raises oxidative activity, which, when combined with a weak antioxidant defense, results in an excess of ROS in mitochondria, damaging other macromolecules like proteins, lipids, and nucleic acids [69]. A persistent elevation of plasma free fatty acids (FFAs) from enlarged adipose tissue causes excessive fat accumulation to induce inflammation and oxidative stress in adipose tissue. This, in turn, activates and upregulates the expression of various pro-inflammatory cytokines (TNF-α, IL-1β, and IL-6), aggravating metabolic alterations [70].

Our results have demonstrated the in vitro antioxidant effect of dill and leeks; consistent with the findings of the current study, numerous previous studies have also shown the antioxidant effects of dill and leeks in vivo. In the livers and kidneys of nicotine-exposed rats, Ajarem et al. [71] discovered that dill extract enhanced cellular antioxidant defenses and inhibited the production of pro-inflammatory mediators, LPO, and ROS. This effect was attributed to the dill’s phenol, flavonoid, and alkaloid content. By boosting the activity of important antioxidant enzymes and dramatically lowering levels of NO and MDA, a byproduct of lipid peroxidation, the leek extract improved antioxidant defense mechanisms [47].

#### 3.3.5. Effect of Functional Formulae on the Inflammatory Markers

Serum inflammatory markers (Figure 2) show significant (*p* < 0.05) rises in liver levels of TLR4, TNF-α, IL-β1, and IL-6 in rats fed the WD diet. Rats administered the dill-psyllium or leek-psyllium formula had significantly decreased levels (*p* < 0.05) of liver TLR4, TNF-α, IL-β1, and IL-6. Hepatic lipid excess brought on by defective lipid metabolism causes lipid peroxidation, which in turn causes oxidative stress, inflammation, and fibrosis. Overproduction of ROS causes hepatocytes to release more cytokines, which damages the liver [72]. Suppressing NF-κB in the liver reduces the expression of genes that produce pro-inflammatory mediators. However, increased hepatic lipid levels have been linked to active NF-κB and increased production of TNF-α, IL-1β, and IL-6 [73]. TNF-α is responsible for several intracellular signaling systems, including the activation of NF-κB, and has been linked to increased activity of Jun N-terminal kinase, which is critical in raising hepatic insulin resistance [74]. Furthermore, because TNF-α is involved in hepatic fatty acid synthesis, increases serum triglyceride levels, activates the liver’s production of VLDL, and promotes hepatocyte cell death and proliferation, it has been reported to play a crucial role in the pathophysiology of liver fibrosis [75].

TLR4 is a crucial receptor on liver cells that activates downstream NF-κB signaling pathways to cause the release of inflammatory cytokines [76]. Since TLR4 suppression might reduce liver damage, it is a potential therapeutic target [77]. In the current study, the dill-psyllium or leek-psyllium formula decreased levels of liver TLR4 and cytokines. This may be attributed to the active phytochemical substances found in dill and leeks that help lower oxidative stress, reduce inflammation, and block inflammatory pathways like NF-kB. Jalili et al. [78] and Xie et al. [12] reported that the proanthocyanidin and phenolic flavonoid content in dill and leek may be responsible for their antioxidant and anti-inflammatory activities. Gallic acid exhibits notable anti-inflammatory properties by activating the NF-κB signaling pathway [79]. A study by Kim et al. [80] suggested that catechins may regulate TLR4 and NF-κB signaling pathways; therefore, the consumption of dill and leeks may contribute to mitigating the inflammatory response due to their catechin and gallic acid content.

#### 3.3.6. Regulatory Mechanism of the Nrf2/Ho-1 Pathway

As shown in Figure 3, in liver tissue, qRT-PCR revealed a significant decrease in the mRNA expression level of Nrf2 in the WD group compared to the normal control group (*p* < 0.05). Similarly, the mRNA expression level of Ho-1 decreased in the WD group compared to the normal control group, whereas NF-κB expression increased in the WD group in comparison to the CN group. Compared to the WD group, the dill-psyllium administration considerably (*p* < 0.05) reduced the expression of NF-κB gene and increased Nrf2 and Ho-1 expression. Similarly, leek-psyllium administration considerably (*p* < 0.05) reduced the expression of the NF-κB gene and increased Nrf2 and Ho-1 expression. One important route for reducing oxidative stress is the Nrf2/HO-1 pathway. By inhibiting RIP3 expression and reducing ROS generation in hepatocytes, activation of this pathway significantly lowers inflammation and fat accumulation. Nrf2 engages in fatty acid metabolism, decreases hepatic fat storage, and suppresses important lipid-synthesizing enzymes as a negative regulator of genes linked to hepatic steatosis. Its activation reduces oxidative stress and fat buildup by promoting lipid breakdown and preventing fat synthesis. By significantly reducing hepatic fat accumulation and halting progression to steatohepatitis, liver fibrosis, and associated conditions, increasing HO-1 enzyme activity may be beneficial in cases of steatohepatitis [81]. Furthermore, by blocking important enzymes like phosphoenolpyruvate carboxykinase and glucose-6-phosphatase, Nrf2 increases hepatic insulin signaling and decreases gluconeogenesis. Additionally, by reducing the expression and activation of liver X receptor-a, SREBP1, fatty acid synthase, and acetyl CoA carboxylase, Nrf2 activators prevented hepatic lipogenesis. Furthermore, by controlling CD36 expression and upregulating PPARα and other β-oxidation-related enzymes, Nrf2 promotes FA absorption and oxidation. Nrf2 has the ability to directly inhibit NF-κB and is regarded as a master anti-inflammatory factor [82]. Nrf2 was discovered to be upregulated by dill and leek. According to Ajarem et al. [71], dill increased hepatic and renal Nrf2/HO-1 signaling and antioxidant defenses, preventing nicotine-induced oxidative damage and inflammation. The Egyptian leek extract activates the Nrf2 pathway, a crucial regulator of cellular antioxidant defense systems, according to Mahmoud et al. [47]. Once activated, Nrf2 enters the nucleus and binds to the antioxidant response element present in the promoter regions of target genes. By inducing the production of antioxidant genes, this binding preserves cellular redox equilibrium and guards against oxidative damage.

#### 3.3.7. Histological Findings

The liver of the control group showed normal hepatic architecture; hepatocytes were arranged in cords radiating from the central veins, with hepatic sinusoids and central vesicular nucleus (Figure 4A). Histopathological examination of the liver sections from the rats that received a high fat-high sucrose diet revealed histopathological changes with loss of regular hepatic architecture and degeneration of hepatocytes. In addition, noticeable microvesicular and macrovesicular steatosis with infiltration of inflammatory cells was also observed (Figure 4B,C). The liver section of the dill-psyllium group showed a moderate ameliorative effect on hepatic architecture and microvesicular steatosis (Figure 4D). On examination of liver section of the leek-psyllium group there improvement with more or less normal hepatic architecture, mild microvesicular steatosis (Figure 4E).

## 4. Conclusions

In rats fed an HF/HS diet, dill-psyllium and leek-psyllium formulations provided significant protection against MASLD and related diseases. Supplementation with the two formulae reduced body weight, fasting blood glucose, insulin levels, leptin levels, cholesterol, triglycerides, LDL-Ch, oxidative markers, inflammatory cytokines, and liver functions, which could be attributed to the effects of the bioactive compounds in each formula. Their activities are associated with the up-regulation of Nrf2 and HO-1 mRNA expression, which improves antioxidant defenses and lessens inflammation. The absence of comparison groups limited to psyllium alone, dill alone, or leek alone, or a positive control group, is considered a limitation of this study. Further future studies are needed, conducted on the ingredients of each formula separately, as well as in vitro studies. Future investigation incorporating hepatocyte models, protein-level analyses, and pathway-specific functional assays would be valuable to further validate the mechanisms suggested by our current gene-expression findings. Additionally, further studies measuring effects on the gut microbiota, safety, and human efficacy are required.

## Figures and Tables

**Figure 1:**
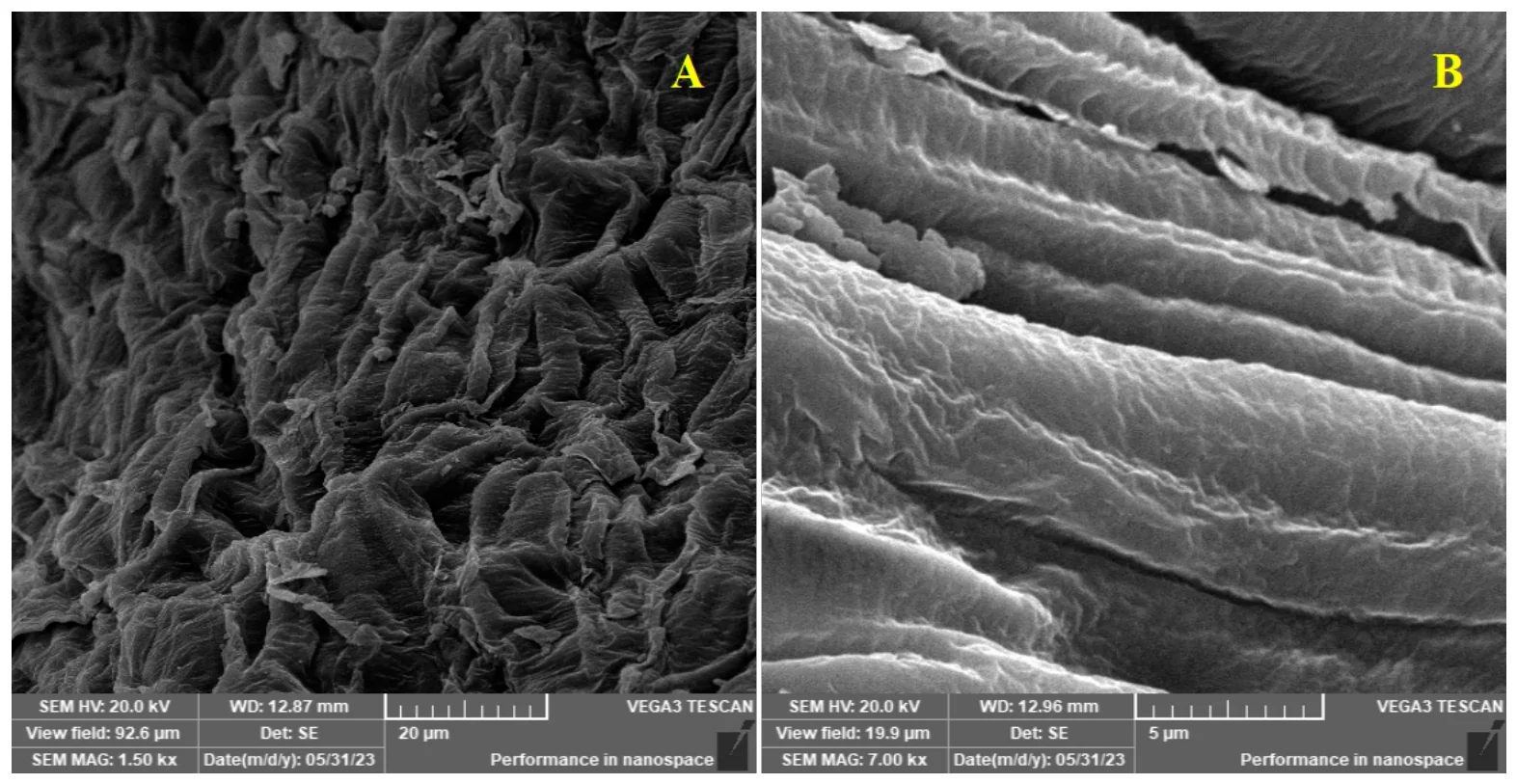
SEM micrographs of psyllium fiber: (**A**) 1.50 k× magnification; (**B**) 7.00 k× magnification.

**Figure 2:**
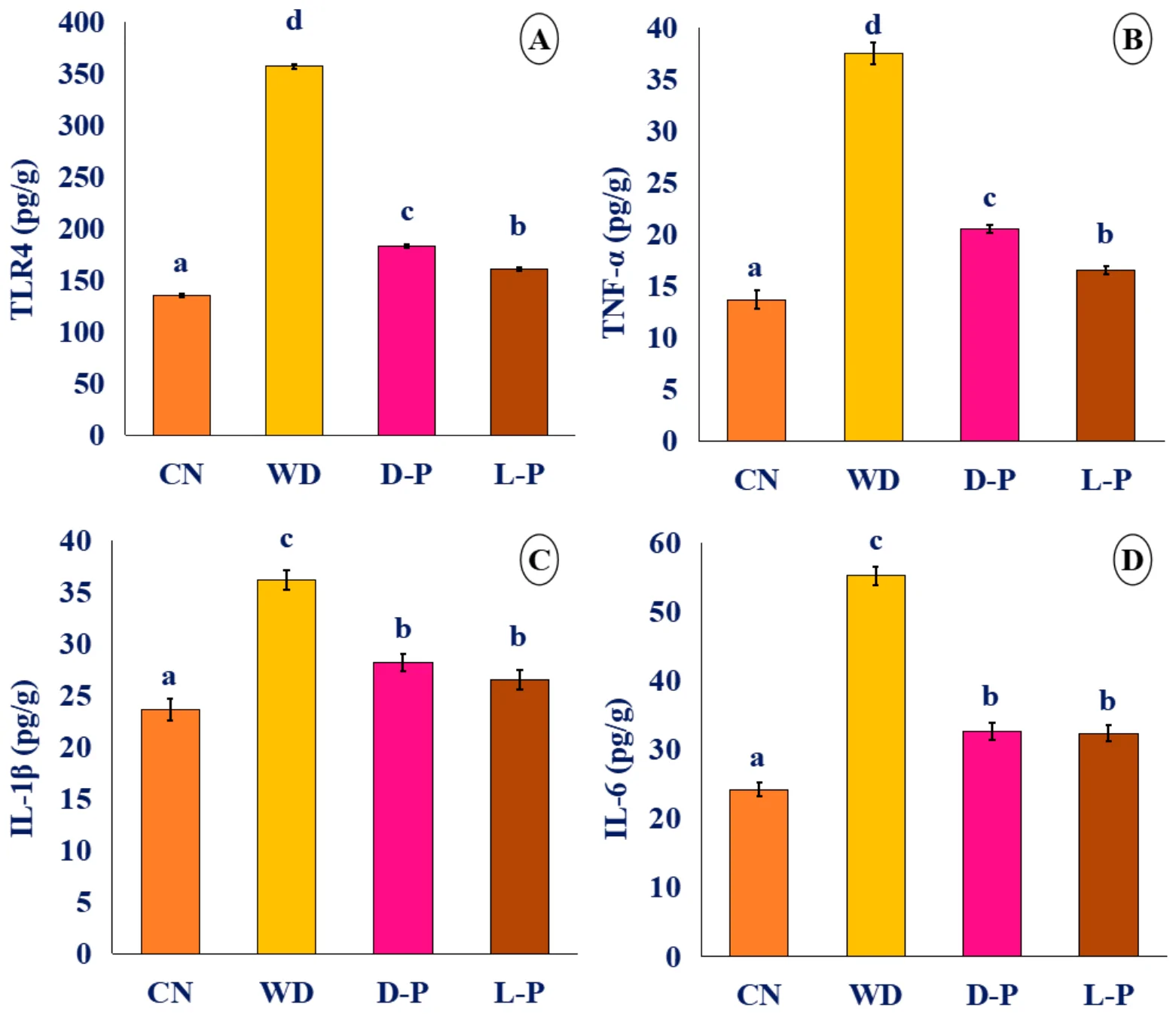
Inflammatory markers. (**A**) TLR4; (**B**) TNF-α; (**C**) IL-1β; (**D**) IL-6. All data are presented as the mean ± SEM (*n* = 6). According to Duncan’s test results, there are significant differences (*p* < 0.05) between the mean values with different letters (a < b < c < d). The normal control group is denoted by CN, the Western diet group by WD, the dill-psyllium group by D-P, and the leek-psyllium group by L-P.

**Figure 3:**
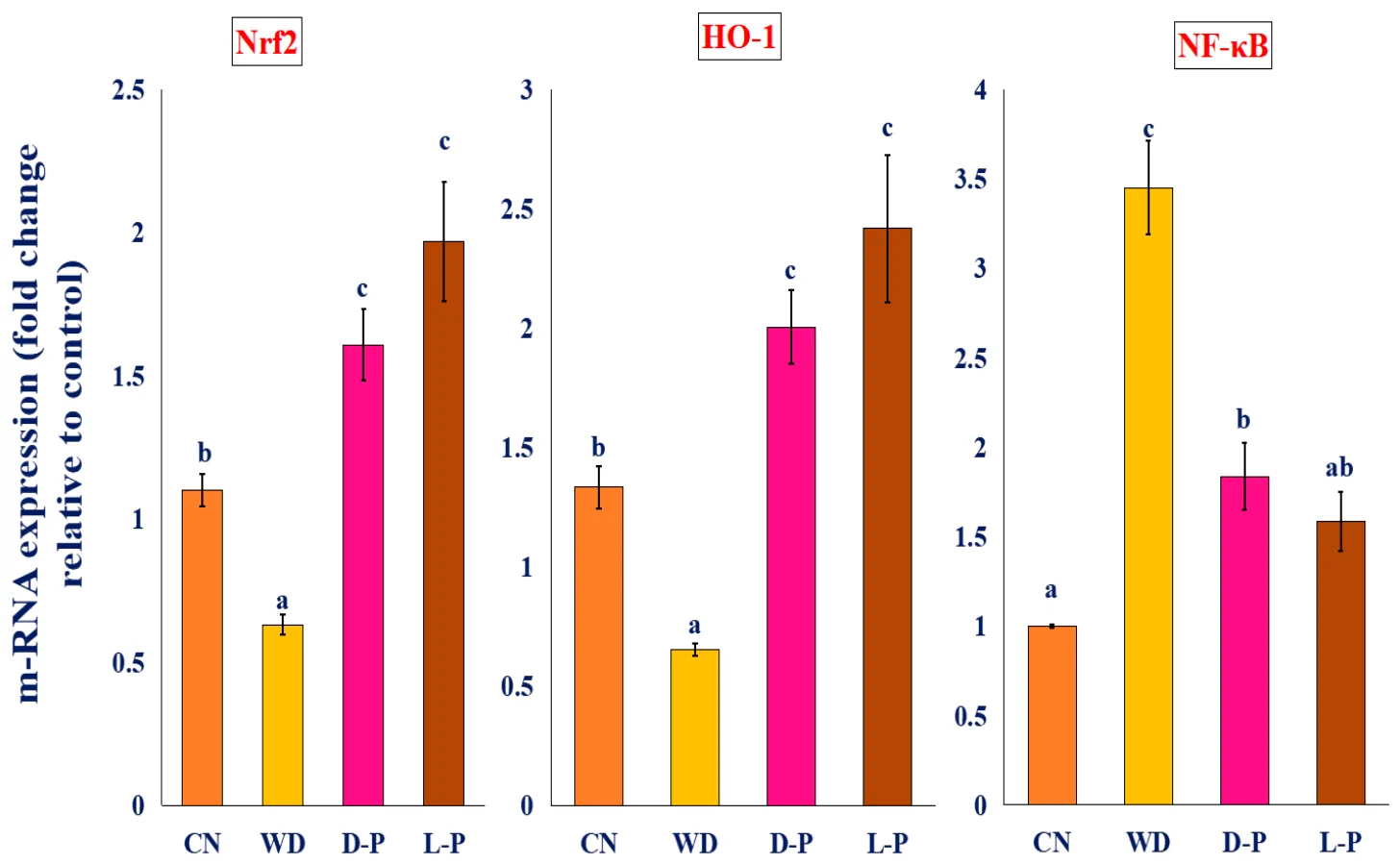
Gene expression of Nrf2, Ho-1, and NF-κB. All data are presented as the mean ± SEM (*n* = 6). According to Duncan’s test outcome, there are significant differences (*p* < 0.05) between the mean values with different letters (a < ab < b < c). The normal control group is denoted by CN, the Western diet group by WD, the dill-psyllium group by D-P, and the leek-psyllium group by L-P.

**Figure 4:**
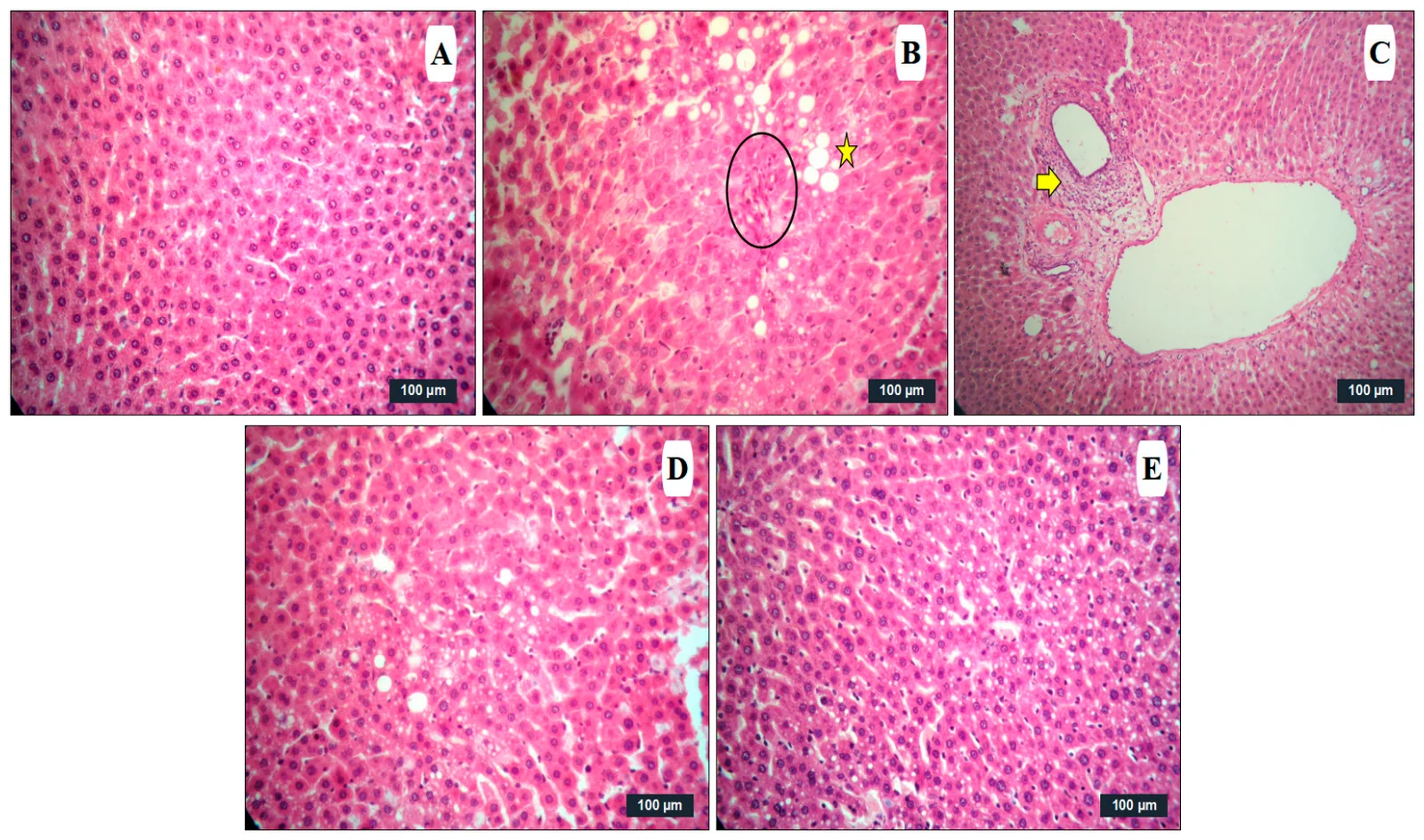
The histopathologic micrographs of the liver tissues (stained with H&E, 200×): (**A**) control group, (**B**) Western diet group with macrovesicular vacuoles (star), (**C**) Western diet group with dilatation of the central vein with inflammatory cell infiltration (arrow) and focal inflammatory cell, (**D**) dill with microvesicular vacuoles, and (**E**) leek group with microvesicular vacuoles.

**Table 1:** The linear gradient of the mobile phase in the determination of water-soluble vitamins.

Time (min)	A (%)	B (%)	Flow (mL/min)
0	90	10	1.5
1	70	30	1.5
4	50	50	1.5
8	90	10	1.5
10	90	10	1.5

**Table 2:** The linear gradient of the mobile phase in the profiling of phenolic compounds.

Time (min)	A (%)	B (%)	Flow (mL/min)
0	82	18	0.9
0–1	82	18	0.9
1–11	75	25	0.9
11–18	60	40	0.9
18–22	82	18	0.9
22–24	82	18	0.9

**Table 3:** The composition of diets (g/100 g).

Ingredients	Balanced Diet	Western Diet
Starch	58.5	27.5
Corn oil	10	20
Fiber	5	1
Sucrose	10	35
Casein	12	12
Salt mixture	3.5	3.5
Vitamins mixture	1	1

**Table 4:** Primers sequence used in the RT-PCR analysis.

Gene	Forward	Reverse	Ref. Seq. mRNA
Nrf-2 (Nfe212)	(5′-3′) AGCAAGGACTGGAGAAGTTCA-	(5′-3′) TCTGTCAGTTTGGCTTCTGG	NM_031789.2
HO-1 (Hmox1)	(5′-3′) GAGCGTCTGCTCAACATCCA	(5′-3′) GTGTTCCTCTGTCAGCATCA	NM_012580.2
NF-κB (p65 subunit, Rela)	(5′-3′) CTTCTGGGCCATATGTGGAGA	(5′-3′) TCCGGAAACACAGCATTCAG	NM_199267.2
β-actin	(5′-3′) GGAGATTACTGCCCTGGCTCCTA	(5′-3′) GACTCATCGTACTCCTGCTTGCTG	NM_031144.3

**Table 5:** Fatty acid composition of freeze-dried dill and leek (mean ± SD, *n* = 3).

Name	Molecular Formula	RT	Area Sum %	
			Dill	Leek
Caprylic acid	C_8_H_16_O_2_	6.888	0.54 ± 0.01	ND
Myristic acid	C_14_H_28_O_2_	20.646	0.99 ± 0.02	1.14 ± 0.02 *
Pentadecanoic acid	C_15_H_30_O_2_	23.507	ND	0.25 ± 0.01
Palmitic acid	C_16_H_32_O_2_	26.168	36.15 ± 0.15	37.06 ± 0.06 *
Palmitoleic acid	C_16_H_32_O_2_	27.439	0.67 ± 0.02	1.69 ± 0.02 *
Heptadecanoic Acid	C_17_H_34_O_2_	28.991	ND	0.49 ± 0.03
Stearic acid	C_18_H_36_O_2_	31.554	19.85 ± 0.03 *	15.5 ± 0.05
Oleic acid	C_18_H_34_O_2_	32.358	4.56 ± 0.01 *	2.47 ± 0.01
Linoleic acid	C_18_H_32_O_2_	34.072	10.48 ± 0.05	16.33 ± 0.1 *
Linolenic acid	C_18_H_30_O_2_	36.195	19.25 ± 0.04	21.33 ± 0.03 *
Arachidic acid	C_20_H_32_O_2_	36.612	0.83 ± 0.02	0.87 ± 0.04
Homo-γ-linolenic acid	C_20_H_34_O_2_	40.105	0.82 ± 0.03	ND
Behenic acid	C_22_H_44_O_2_	41.395	1.07 ± 0.03 *	0.77 ± 0.01
Erucic acid	C_22_H_42_O_2_	41.96	2.99 ± 0.01 *	0.26 ± 0.01
*cis*-13,16-Docosadienoic acid	C_22_H_40_O_2_	43.366	1 ± 0.03 *	0.81 ± 0.02
Lignoceric acid	C_24_H_48_O_2_	45.892	0.79 ± 0.02 *	0.59 ± 0.04
Nervonic Acid	C_24_H_46_O_2_	48.109	ND	0.44 ± 0.01

* According to the *T*-test results, there are significant differences (*p* < 0.05). ND; not detected.

**Table 6:** Vitamin quantification of freeze-dried dill and leek (mean ± SD, *n* = 3).

	Dill	Leek
	Conc. (µg/g)	
Vitamin A	25.9 ± 0.05 *	13.73 ± 0.2
Vitamin E	ND	5.43 ± 0.03
Thiamine (B1)	54.793 ± 0.1 *	29.983 ± 0.06
Riboflavin (B2)	34.840 ± 0.12 *	6.971 ± 0.02
Niacin (B3)	439.086 ± 1.01 *	187.091 ± 1.0
Pyridoxine (B6)	749.138 ± 0.3 *	362.162 ± 0.03
Folate (B9)	90.164 ± 0.03 *	27.413 ± 0.01

* According to the *T*-test results, there are significant differences (*p* < 0.05). ND; not detected.

**Table 7:** Phenolic compounds of freeze-dried dill and leek (mean ± SD, *n* = 3).

	Dill	Leek
	Conc. (µg/g)	
Gallic acid	878.39 ± 1.1 *	151.84 ± 0.2
Chlorogenic acid	7370.94 ± 2.01 *	15.88 ± 0.07
Catechin	1528.47 ± 0.2 *	286.36 ± 0.03
Methyl gallate	ND	196.68 ± 0.03
Caffeic acid	52.22 ± 0.21 *	28.31 ± 0.01
Syringic acid	82.61 ± 0.1 *	3.14 ± 0.02
Pyrocatechol	47.26 ± 0.2	ND
Rutin	53.31 ± 0.2	927.46 ± 0.05 *
Ellagic acid	4.69 ± 0.3	17.73 ± 0.03 *
Coumaric acid	3.26 ± 0.02	9.77 ± 0.05 *
Vanillin	8.66 ± 0.4	52.03 ± 0.02 *
Ferulic acid	9.44 ± 0.04	41.47 ± 0.04 *
Naringenin	17.96 ± 0.02	71.02 ± 0.01 *
Rosmarinic acid	4.16 ± 0.01	811.30 ± 0.2 *
Daidzein	1.99 ± 0.005	4.17 ± 0.01 *
Quercetin	37.10 ± 0.1	575.53 ± 0.09 *
Cinnamic acid	25.87 ± 0.11	35.66 ± 0.05 *
Kaempferol	7.19 ± 0.04	269.82 ± 0.1 *
Hesperetin	14.68 ± 0.01	34.80 ± 0.3 *

* According to the *T*-test results, there are significant differences (*p* < 0.05). ND; not detected.

**Table 8:** TPC, TFC, and antioxidant properties of freeze-dried dill and leek (mean ± SD, *n* = 3).

	Dill	Leek
TPC (mg GAE/g)	137.43 ± 1.10 *	121.17 ± 2.04
TFC (mg CE/g)	44.23 ± 1.46 *	35.60 ± 1.22
DPPH inhibition %	78.20 ± 1.01 *	65.10 ± 0.85
ABTS inhibition %	52.27 ± 1.10 *	43.77 ± 1.66
FRAP (µmol Fe(II) equivalent/g)	56.23 ± 1.20 *	35.90 ± 1.31

* According to the *T*-test results, there are significant differences (*p* < 0.05).

**Table 9:** Growth performance parameters.

	CN	WD	D-P	* Change (%)	L-P	* Change (%)
Initial Body Weight (g)	133.33 ± 2.14 ^a^	133.83 ± 1.45 ^a^	133.33 ± 1.89 ^a^	0	133.83 ± 1.81 ^a^	0
Final Body Weight (g)	208.17 ± 2.88 ^a^	234.83 ± 5.40 ^b^	208.50 ± 3.51 ^a^	11.21	205.50 ± 1.91 ^a^	12.49
Body Weight Gain (g)	74.83 ± 4.84 ^a^	101.00 ± 5.57 ^b^	75.17 ± 4.29 ^a^	25.57	71.67 ± 2.42 ^a^	29.04
Total Food Intake (g)	828.67	839.67	787.67	6.19	785.67	6.43
Fat Weight (g)	5.98 ± 1.04 ^a^	8.72 ± 0.85 ^b^	5.94 ± 0.72 ^a^	31.88	5.45 ± 0.20 ^a^	37.50
Liver Weight (g)	5.97 ± 0.22 ^a^	7.49 ± 0.67 ^b^	6.21 ± 0.25 ^a^	17.09	6.09 ± 0.15 ^a^	18.69

All data are presented as the mean ± SEM (*n* = 6). According to Duncan’s test results, there are significant differences (*p* < 0.05) between the mean values with different letters (a < b). The normal control group is denoted by CN, the Western diet group by WD, the dill-psyllium group by D-P, and the leek-psyllium group by L-P. Total food intake was calculated for a group of rats housed in cages. * is estimated relative to the WD group, and these data are descriptive only.

**Table 10:** Biochemical results.

	CN	WD	D-P	* Change (%)	L-P	* Change (%)
FBG (mg/dL)	81.33 ± 1.72 ^a^	142.83 ± 2.27 ^d^	106.50 ± 2.20 ^c^	25.43	92.83 ± 2.70 ^b^	35.01
Insulin (U/L)	4.75 ± 0.20 ^a^	6.87 ± 0.15 ^c^	5.50 ± 0.22 ^b^	19.94	4.98 ± 0.23 ^ab^	27.51
Leptin (ng/mL)	0.61 ± 0.06 ^a^	3.50 ± 0.22 ^c^	1.38 ± 0.06 ^b^	60.57	1.15 ± 0.09 ^b^	67.14
AST (U/L)	49.83 ± 0.75 ^a^	82.50 ± 0.92 ^c^	59.33 ± 1.28 ^b^	28.08	56.17 ± 1.62 ^b^	31.91
ALT (U/L)	41.50 ± 1.28 ^a^	76.17 ± 1.25 ^c^	48.83 ± 0.87 ^b^	35.89	44.33 ± 0.33 ^a^	41.80
ALP (U/L)	148.17 ± 2.09 ^a^	253.33 ± 2.15 ^c^	160.50 ± 1.33 ^b^	36.64	152.83 ± 1.35 ^a^	39.67
Fat in liver (mg/g tissue)	17.83 ± 0.60 ^a^	49.50 ± 1.48 ^c^	24.83 ± 1.40 ^b^	49.84	21.67 ± 0.88 ^b^	56.22

All data are presented as the mean ± SEM (*n* = 6). According to Duncan’s test results, there are significant differences (*p* < 0.05) between the mean values with different letters (a < b < c < d). The normal control group is denoted by CN, the Western diet group by WD, the dill-psyllium group by D-P, and the leek-psyllium group by L-P. * is estimated relative to the WD group, and these data are descriptive only.

**Table 11:** Lipid profile parameters.

	CN	WD	D-P	* Change (%)	L-P	* Change (%)
Total Cholesterol (mg/dL)	90.67 ± 2.09 ^a^	142.17 ± 1.80 ^d^	116.33 ± 1.14 ^c^	18.17	102.50 ± 1.06 ^b^	27.90
Triglycerides (mg/dL)	83.33 ± 1.63 ^a^	156.50 ± 0.99 ^c^	98.50 ± 1.94 ^b^	37.06	94.33 ± 1.72 ^b^	39.72
LDL-Cholesterol (mg/dL)	42.33 ± 1.11 ^a^	87.00 ± 1.50 ^d^	59.33 ± 0.71 ^c^	31.80	51.00 ± 0.57 ^b^	41.38
HDL-Cholesterol (mg/dL)	39.50 ± 0.43 ^d^	27.83 ± 0.60 ^a^	33.33 ± 1.08 ^b^	−19.76	35.83 ± 0.70 ^c^	−28.75
Cholesterol/HDL ratio	2.30 ± 0.05 ^a^	5.12 ± 0.10 ^d^	3.51 ± 0.12 ^c^	31.44	2.87 ± 0.07 ^b^	43.94
Non-HDL Cholesterol (mg/dL)	51.17 ± 1.99 ^a^	114.33 ± 1.69 ^d^	83.00 ± 1.48 ^c^	27.40	66.67 ± 1.41 ^b^	41.69
VLDL (mg/dL)	16.67 ± 0.32 ^a^	31.30 ± 0.20 ^c^	19.70 ± 0.39 ^b^	37.06	18.87 ± 0.34 ^b^	39.71

All data are presented as the mean ± SEM (*n* = 6). According to Duncan’s test results, there are significant differences (*p* < 0.05) between the mean values with different letters (a < b < c < d). The normal control group is denoted by CN, the Western diet group by WD, the dill-psyllium group by D-P, and the leek-psyllium group by L-P. * is estimated relative to the WD group, and these data are descriptive only.

**Table 12:** Oxidative stress markers.

	CN	WD	D-P	* Change (%)	L-P	* Change (%)
TAC (mmol/L)	2.95 ± 0.07 ^b^	1.32 ± 0.06 ^a^	2.75 ± 0.08 ^b^	−108.33	2.95 ± 0.06 ^b^	−123.48
ROS (pg/g)	56.50 ± 0.99 ^a^	199.33 ± 1.43 ^d^	74.17 ± 1.42 ^c^	62.79	68.50 ± 1.78 ^b^	65.63
MDA (nmol/g tissue)	10.83 ± 0.48 ^a^	21.67 ± 0.88 ^c^	14.33 ± 0.49 ^b^	33.87	13.33 ± 0.67 ^b^	38.49
SOD (U/g tissue)	4.95 ± 0.07 ^c^	2.15 ± 0.08 ^a^	4.18 ± 0.08 ^b^	−94.42	4.37 ± 0.16 ^b^	−103.25
GSH (mg/g tissue)	23.50 ± 0.85 ^c^	14.50 ± 0.99 ^a^	19.17 ± 0.60 ^b^	−32.21	19.67 ± 0.84 ^b^	−35.65

All data are presented as the mean ± SEM (*n* = 6). According to Duncan’s test results, there are significant differences (*p* < 0.05) between the mean values with different letters (a < b < c < d). The normal control group is denoted by CN, the Western diet group by WD, the dill-psyllium group by D-P, and the leek-psyllium group by L-P. * is estimated relative to the WD group, and these data are descriptive only.

## Data Availability

The original contributions presented in this study are included in the article. Further inquiries can be directed to the corresponding author.
